# Application of Magnetic Sensor for Magnetic Profile (1D) and Surface (2D) Measurement of Automotive Wheels

**DOI:** 10.3390/s21072475

**Published:** 2021-04-02

**Authors:** Sebastian Brol

**Affiliations:** Department of Vehicles, Opole University of Technology, ul. Prószkowska 76, 45-758 Opole, Poland; s.brol@po.edu.pl; Tel.: +48-77-449-8000

**Keywords:** magnetic flux density sensor, tire, measurement device

## Abstract

This paper shows a report of over three years of intensive work on application of a 3-axis anisotropic magnetoresistive sensor with I^2^C interface for measurement of magnetic flux density distribution of automotive wheels. The work was undertaken to answer the question of whether is a possibility to effectively apply low-cost magnetic sensors with serial interface to measure the magnetic field surrounding the automotive wheel or tire. Two measurement techniques were discussed: Magnetic profile (1D) and magnetic surface measurement (2D) over tread, and also gear associated with the sensor, as well as its design, layout, operation, and control technique during (1D) and (2D) measurements. Three experiments were performed to asses accuracy and repeatability concerning component and resultant magnetic circumferential profiles and also magnetic surface. Differences between measurement outcomes in experiment were assessed. The results show that accuracy and repeatability lays below maximum admissible uncertainty declared by the producer. This proves directly that there is no measurable influence of motors, gear, operation, or measurement procedure on results obtained by magnetic sensors, and indirectly, that the assumed requirements regarding gear design and parameters are correct, and measurement of magnetic flux density distribution of automotive wheels and tires using (1D) and (2D) techniques is possible using a 3-axis anisotropic magnetoresistive sensor with I^2^C interface.

## 1. Introduction

**Figure 1:**
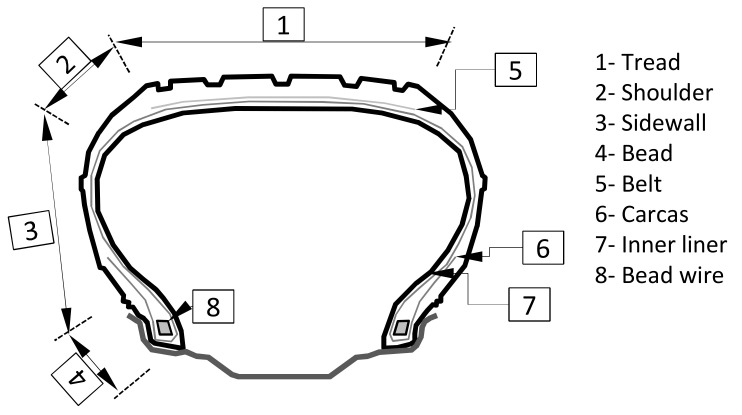
Cross-section of tire.

The automotive tires shown in Figure 1 (especially those fixed in passenger cars and trucks) consist of many elements with different utilitarian functions. Some parts of the tire (tread, arm, side) are used to assure tire flexibility and damping properties, and the others (like steel belt/belts, cords) provide stiffness and shape preservation under constant and variable loads [1,2].

In mass production, a wide range of available materials is used to manufacture tire components with preservation of overall tire quality. Therefore, it is also with the so called steel belt, which consists among others of iron alloys. Its name is misleading because it is not a belt, but rather few layers of over-eutectic steel wires of diameter varying from 0.1 mm up to 0.5 mm. In every layer, the steel wires are arranged parallel to each other and embedded in rubber layer. Typically, two such layers placed one above the other (but can be more) are used in tire construction. The wires in one layer are arranged at an angle to the second one. This creates a structure which resembles a mesh. The “steel belt” term comes rather from fact that this element acts as a kind of belt, which buckles the tire insides and keeps it in shape. The steel belt structure is complicated, but durable and strong, and therefore it is placed beneath of the tire’s tread. The deflection of the tire during the car’s forward movement depends mainly on static and dynamic load, and the tire’s radial stiffness, which is related to pumping pressure, temperature, and physical properties of materials used for its construction and tire’s elements arrangement and dimensions [3,4,5].

During rotation, the belt deflects similarly to the tread. The belt’s deflection is caused by many factors like: Pumping and actual pressure, wheel rotation, car cornering, accelerating, braking, obstacle traversing. All of these factors are undergoing perpetual change with tire age, mileage, and wear. According latest investigations the belt’s magnetic field distribution and it’s intensity also changes in time during normal exploitation. Therefore, it can be presumed that the process which magnetizes the wires in the steel belt is the reverse magnetostriction inducted by stresses. The magnetic field in the steel belt is an remaining artifact of many processes happening during tire exploitation. The measurement of its distribution or value itself does not reveal these properties. They must be extracted from measurements using signal analysis technique (which is out of the scope of this article). At this moment, it is vital to find out what process creates which magnetic field distribution, and after that to determine or estimate some physical characteristics of the tire. To finalize this vital part of work, first a proper measuring technique, as well as measuring approach, gear, and equipment, must be selected. These are the subjects discussed in this the article. This task is quite demanding because a tire with a steel belt during normal exploitation rotates (together with the rim) in Earth’s magnetic field. It can be assumed that Earth’s induction is uniform and its intensity varies from 25 µT to 60 µT [6,7]. The rotation frequency of the wheel can vary from 0 Hz to even 50 Hz in case of sport cars [8,9]. The combined influence of the earlier mentioned factors and additional taking into account of the steel belt’s deflection at every wheel rotation is enough to create circumstances for the Vilary effect, which can change both the steel belt’s magnetic field intensity [10] and its distribution [9]. The changes can be significant [7,10,11,12], therefore a repeatable circumstances for measurements must be created. Therefore, beside sensor selection also proper measurement technique as well as measuring instrument design and action is required.

As mentioned before, measuring techniques must be consciously selected for the measurement device or fixture. The earlier work of researchers documents ingenious approaches to this topic. Stankowski [10], Jacobs et al. [13], and Kawase et al. [14,15] exclusively used handheld instruments or instruments combined with special or improvised fixtures intended to fix and rotate the complete wheel (rim + tire). Such measurements give the information about the magnetic profile course, but the measurement result (including profile) is rather difficult to repeat. The measurements in all cases are made in time domain and after that analyzed [16,17]. Earlier, the scientists used the wooden fixtures for rotating the wheel. Optimally, the wheel balancing machines were used for this purpose, but it was commonly made of ferromagnetic material such as steel, and therefore interfered with the measurement results. The authors were aware of the influence of ferromagnetic materials on the measurement results since the investigations were considered as preliminary. Often (and rightly), the influence of magnetic materials of fixtures was assumed to be negligible as it follows among others from [18,19], but only in case of very close proximity of the sensor to the tire’s tread.

A different approach was presented by Brol et al. in [9], where the measurement was made in angle of rotation domain on measurement device with controlled kinematic parameters (angular speed), but still loaded with some repeatability error because of influence of human factor during sensor position setting in the direction of tire’s tread width.

There was many standalone sensors (also the low-cost) described among others in 1819, but this paper concerns the application of 3 axis magnetic sensor with needed gear to provide repeatable and possibly accurate measurement of magnetic profile (1D) and surface (2D). The profile and surface are measured above steel belt (and tread) in automotive tire.

## 2. Materials and Methods

### 2.1. The Sensor

For measurement the 3-axis anisotropic magnetoresistive HMC5883L sensor was selected [20]. It was chosen because of sufficient, selectable range, low price, and digital data transmission via I^2^C interface. The maximum sampling frequency is 160 Hz (for 3 16-bit composite of B). The sensor’s overall resolution and accuracy is 0.48 × 10^−7^ T and 2 × 10^−7^ T accordingly.

### 2.2. Measurement Method

**Figure 2:**
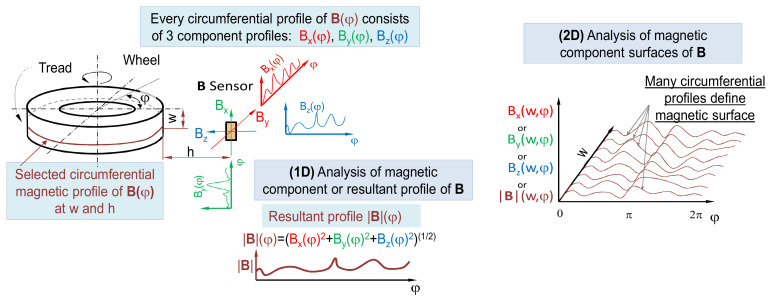
Schematic of magnetic circumferential profile measurement (1D) and magnetic plane measurement (2D).

Nowadays there are two main methods of the tire’s magnetic field measurement over the tire’s tread (and steel belt), as depicted in Figure 2. The first one is the measurement of the magnetic profile along tread’s circumference. This is so-called (1D) measurement [21], and the result is a circumferential profile of vector **B**. The **B** vector can consist of many projections of **B** in dependence of sensor design. The sensor itself can be equipped in one, two, or three circuits for measuring appropriate Cartesian projections of **B** like, for example, *Bx*, *By*, *Bz*. Therefore, the circumferential magnetic profile can refer to one of four profiles describing change of *Bx*, *By*, *Bz*, and |**B**| along the wheel’s circumference at the selected tread’s width.

The second measurement method is the (2D) spatial scan over tire’s tread surface. The scan is usually made on an area defined by circumference of the tire and (usually) width of the tread. The result of such a measurement is the so-called magnetic surface. For practical reasons, the magnetic surface consists of a finite number of circumferential profiles measured following each other tread’s width.

**Figure 3:**
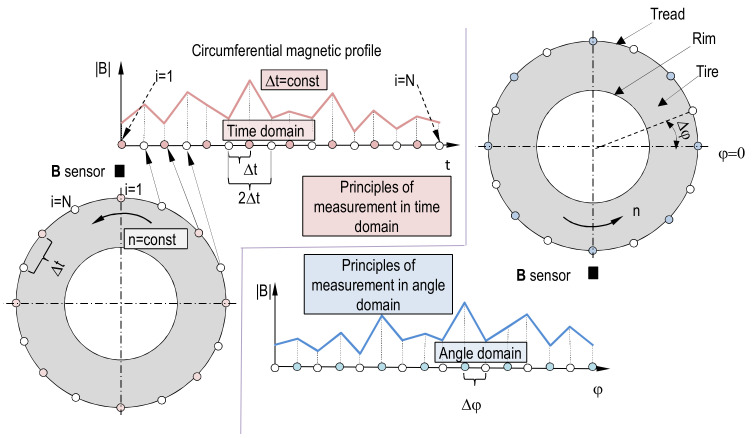
Schematic of two methods of measuring of circumferential magnetic profile (1D): In time domain and in angle domain. In this investigation, the Δφ method is utilized.

In this particular situation, both the problems of accuracy and repeatability of magnetic profile measurement appear. These problems must be solved by applying, simultaneously, mechanical, electronic, and informatic solutions fulfilling the requirements defined further. The magnetic plane, which consists of many circumferential profiles, requires that every magnetic profile element should be measured at the same point on circumference as the same elements of profiles, which create the magnetic plane. Additionally, there are two techniques of measuring magnetic circumferential profile in two domains: The time and the angle domain, as shown in Figure 3.

In earlier investigations, measurement of profile in time domain were utilized. It is often observed that the same profile measured several times in time domain can vary significantly if angular speed or sample rate are not maintained constant. To perform a repeatable measurement magnetic profile and consequently magnetic surface, a more reliable method is recommended.

Measurement of B in angle domain provides a solution to this problem, as depicted in Figure 3. The measurement is triggered by an angle measurement device (like a microcontroller, impulse, or absolute encoder) at a demanded angle increment or at a desired angular position. The trigger engages every time for the same angle, but the real angle of circumferential profile measurement depends on the moment of B measurement after the trigger event and this depends on rotational speed, the trigger latency, and code execution speed. To minimize discrepancy between real angle of measurement and angle of triggering the rotational speed must be kept both low and constant. These requirements bring a potential risk of stick and slip phenomena in bearing mechanisms. This phenomena can be beaten by increase of speed, but the angular velocity cannot be too high because of centrifugal force which can cause vibrations inducted by uneven mass distribution [22,23,24]. Therefore, the time between two **B** samples measurement *t_s_* should not exceed the value given by Formula (1). Additionally, according to Formula (1), *t_s_* was set assuming *x* = 1.1, *ω* = 1.2 rad/s, *TRGs* = 1024.

(1) $$t_{s}=\frac{2\pi }{x\cdot \omega \cdot TRGs}$$

where: *t_s_*—time of execution of **B** sample measurement, *x*—assumed safety coefficient, advised *x > 1*, *TRGs*—count of assumed measurement of B triggers during one rotation, *ω*—angular speed in rad/s.

### 2.3. Mechnical Design

The measurement device is designed as low-height object. The reason for that is to prevent the operator from lifting on significant height heavy tires and wheels. If access to a tire’s tread is needed, the device can be put on a wooden desk or base. The measuring device is constructed using materials which are not influencing the magnetic field *µ_r_* = 1.000012 to *µ_r_* = 1.05 (such as alumina, brass alloys, and stainless austenitic steel of grade given in Table 1), yet still show good hardness and stress resistance on loads related with acceleration and deceleration of tested wheel without any excessive material addition.

The automotive complete pneumatic wheel in general consists of a rim, which can be made either from iron, alumina, or magnesium based alloys, and the tire itself, which is made of rubber, iron alloy wires, and artificial fibers like aramids, polyamide, and others. The ferromagnetic materials can be found in the wheel rim, wire rim, and steel belt. The engineering materials which this measurement device is made of must be durable, stress-resistant, and should have magnetic permeability as close as possible to vacuum in order to not interfere with the magnetic field of wheel (not to “amplifying” it). Ferromagnetic alloys must be avoided because of it high relative permeability but austenitic steels, aluminum alloys, and brass as well as artificial materials can be used. These materials are usable mainly because of low relative permeability, which is not greater than *µ_r_* = 1.020 (Table 1) and because of sufficient yield stress. In the second version of measuring device, which is described in this paper, austenitic steel 316 grade, common brass (63% Cu, 37% Zn), and aluminum alloy AlCu_4_MgSi(A) was used.

**Figure 4:**
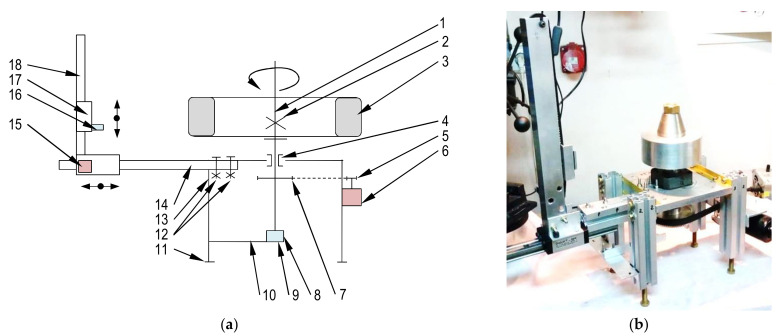
Original measurement device schematic (**a**). 1—main shaft, 2—fastening nut, 3-wheel, 4—bearing, 5,7—sprockets, 6—electric motor, 8—encoder, 9—encoder’s base plate, 10—arm, 11—foot, 12—locking screws, 13—leg, 14—guide, 15—electric motor, 16—sensor, 17—slide, 18—pol. (**b**) Original photo of measuring device.

As a final result of considerations and investigations, the kinematic diagram and real photo of the measuring device are shown in Figure 4a,b, respectively. The basic parameters of the device are given in Table 2.

The shaft on Figure 4, detail 1, which rotates the wheel relative to the sensor was shortened in length, therefore a decrease of mass and dimensions was achieved by preserving the stiffness. The fixing of the wheel to the shaft is assured by friction enhanced by force excreted by fastening a nut fixed on the threated end of the shaft.

### 2.4. Measurement Dynamics

To prevent it from exceeding the friction force by rapid acceleration, the shaft was accelerated with a constant acceleration equal to 0.1 rad/s^2^. This generates torque equal to 0.3 Nm for the wheel with I = 3 kg m^2^. The opposition friction torque for the lightest considered wheel of mass 18 kg is 0.4 Nm (the heavier the wheel, the better, because inertia “tries to move the wheel” and the wheel’s mass combined with friction prevents it from happening). This means, that the wheel’s mass is enough to assure proper measurement operation. The friction torque can be further increased by screwing in the fastening the nut.

The electric motor with 1:62 reduction gear powers the shaft through timing belt gear of ratio equal to 1:10. The electric motor is shielded and distant from the wheel as well as the B sensor.

The sensor itself is fixed to a slide trolley, which moves vertically along a vertical guide. The vertical guide is fastened by bolts to a horizontal trolley on a horizontal guide bar. The horizontal trolley is operated manually in order to set the sensor’s distance from tire’s tread. The vertical lift uses a stepper motor to change the sensor location at the tire’s tread width. The stepper motor of the vertical lift, because of its close proximity to the sensor, is not powered during circumferential profile measurement. This is due to a need to minimize disturbances. Moreover, at the beginning the measurement procedure for the ambient magnetic field, compensation is executed.

### 2.5. Electric Currents and Motor Magnetism

The measuring device’s shaft must be powered to provide torque needed for rotating the wheel. The easiest way is to use an electric motor for that purpose, but the motor itself must be either magnetically shielded or placed at a considerable distance to the wheel and the sensor. The power cables must be also distant or shielded. This setup assumes that the magnetic field inducted by motor during operation will be lower or equal to sensor resolution at sensor location and also weak enough to not induce additional magnetic field (at tire’s steel rim, bead wires, and steel belt) which can interfere with measurements results.

For this particular measuring device, the electrical currents were reduced because of the use of permanent magnets direct current (PMDC) electric motor with worm gear, the power cables are shielded and distant from the wheel and sensor. Additionally, the motor is shielded by an aluminum plate from the tire and rim, and also distant from the wheel by use of a timing belt drive.

For this motor (and gear), the electric current is proportional to torque. The current measured by a handheld device in acceleration phase is *i_a_* = 1.24 A, during measurement, *i_m_* = 0.37 A, and during deceleration, *i_d_* = −0.19 A. This is adequate to magnetic flux densities measured 0.6 m from the power cable *B_a_* = 0.07 × 10^−7^ T, *B_m_* = 0.02 × 10^−7^ T, *B_d_* = −0.009 × 10^−7^ T calculated using Biot-Savart law [17,25]. Please note that *B_m_* is significantly lower than the HMC5883L magnetic sensor’s resolution and accuracy, which is 0.48 × 10^−7^ T and 2 × 10^−7^ T accordingly. *B_m_* value is calculated exactly at the beginning of the tire side. The current during measurement (when keeping a constant angular speed by angular speed controller action) is at least 4 times smaller, therefore one can conclude that its operation does not influence the measurement results.

### 2.6. Angular Speed Control

Regardless of measuring either in time domain or in angle of rotation domain, the angular speed must be controlled. The automotive wheel has considerable inertia from *I* = 0.5 kg m^2^ to over *I* = 1.2 kg m^2^ [3], and therefore if the bearings do not generate additional variable resistances to movement and the motor rotates without so called “torque ripples”, then the angular speed control can be omitted. On the other hand, the controller with a too short time constant can force the wheel to slip in the fixture and therefore cause misalignment in relation to the rotation axis of the shaft. Therefore, the controller should be tuned as overdamped (see for reference [26]) and according to parameters calculated by Formulas (2)–(4).

(2) $$t_{c}\geq \frac{0.63\omega 2I}{mgd}$$

(3) $$e_{static}=\int_{t=0}^{t=nt_{s}}edt=0\;(\mathrm{referred}\;\mathrm{to}\;\mathrm{one}\;\mathrm{rotation})$$

(4) $$e_{dynamic}\leq \left|\frac{2\pi }{nt_{s}}\right|$$

where: *e*—control error, *e_static_*—the static part of control error, *e_dynamic_*—the dynamic part of control error, *m*—mass of wheel, *I*—moment of inertia of wheel, *g*—gravity constant, *d*—diameter of contact plane between wheel and fixture, *ω*—angular speed, *n*—number of samples around circumference, *t_s_*—time between sample acquisition of **B**.

For this device, the structure of a PID controller was utilized with parameterized integral and proportional actions in such a way that it fulfills the rules given by Formulas (2)–(4) and not exceed an acceleration or deceleration of 0.1 rad/s^2^.

### 2.7. Software Issues and Data Exchange

The whole control system structure is a system consisting of many microcontrollers. It is because it is beneficial in prototyping phase of complicated device. Especially if it is a “new prototype of its kind”. In such a system, for every single microcontroller, the tasks are relatively simple, and therefore, it is easier to manage the code and also make adaptations and enhancements to which almost all prototypes are subjected to. There are also drawbacks—the microcontroller communication must be “invented”, coded, and implemented on every microcontroller in the system, and the software for any microcontroller must use different resources and namespaces in order to integrate the code in the final stage of product preparation in one microcontroller [27,28]. In this particular case, it is decided to design a microcontroller system consisting of 3 microcontrollers. There are two main reasons for that besides the designer’s convenience. For this device, many improvements of software during the testing phase are expected. It is also crucial to have the possibility to test the efficiency of many algorithms, which should increase accuracy and repeatability of measurement of circumferential profile.

**Figure 5:**
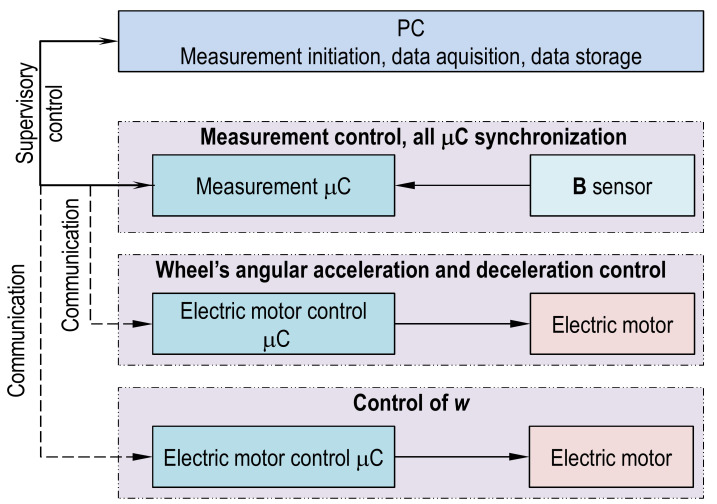
Schematic of microcontroller system layout and primary function of every microcontroller (μC).

Three fundamental tasks were defined in system and presented in Figure 5. They are as follows: **B** samples measurement, wheel acceleration/deceleration control, and sensor location control along tread width. It is important that in measurement mode all the fundamental tasks must be synchronized.

### 2.8. Operation Algorithm

The accuracy and repeatability is the effect of all design decisions made in the area of mechanical design, programing, electric and electronic systems, and also the operation algorithms. In this measuring device, constant magnetic flux density compensation was implemented. The idea is that the ambient magnetic flux density cannot be the same for all sensor locations in the w direction, and it can (potentially) depend on angle of rotation of shaft during executing magnetic scan (2D) or circumferential profile measurement (1D) (see Figure 3 for reference). Therefore, before wheel fixing on the shaft, the static values of **B**_stat_ are determined for all w’s used. The novelty here is that **B**_stat_(w) can be defined both as a constant value or as a function of shaft rotation angle. After measurement, the results are simply subtracted from previously determined **B**_stat_(w) values. The algorithm can be used for circumferential profile measurement (1D) as well as for magnetic scans (2D).

The final parameters of complete measurement device is provided in Table 2.

## 3. Measurement Objects and Measurement Course

To prove the usefulness of application of this sensor with the above described equipment to (1D) and (2D) measurement of the tire’s magnetic field, three tests were made. For all tests, the same winter tires were utilized, fixed on light alloy (alumina alloy) 15″ rims. According to description on its side, their calculated width is 205 mm, height 123 mm. The plies are made of polyamide, and the steel belt consists of 2 layers of steel wires. The tires are exploited from new ones through 2 years in the same vehicle in the same location, and even on the same orientation relative to the hub.

The tests were as follows:

(1) The test of noise of the sensor, when influence of the stopped and running motor which powers the spindle is assessed. The sensor is placed in several distances (*h*_0_) from the rotating shaft and at several widths of the tire tread (w). For every sensor location, 3 magnetic composites of circumferential profiles are measured. The assumption is: If the standard deviation of every circumferential profile is lower than the admissible standard deviation of the sensor, then there is no detectable influence of the gear and equipment operation on the measurement result.
(2) Repeatability test 1, for assessing the discrepancy between measured one after another composite magnetic profiles. This time the wheel is fixed to the shaft. The measurements are conducted at the same tread’s width (w = 100 mm) and sensor’s distance from the tread surface (*h* = 55 mm). There are 10 measurement performed one after another. In every measurement, 3 composite circumferential magnetic profiles are acquired. Every circumferential profile consists of 1024 samples. Profiles 2 to 10 were subtracted from first one in such way that only values for the same sample number are subtracted in the whole profile. Finally, 9 × 3 composite profiles of differences are obtained. The assumption is: If the highest standard deviation of every of 9 × 3 profiles of differences is lower than the admissible standard deviation of the sensor, then there is no detectable influence of the gear and equipment in this mode of operation.
(3) Repeatability test 2, for assessing the discrepancy between the measured one after another magnetic composite surface. The wheel is fixed to the shaft. The measurements are conducted at the same sensor distance from the tread’s surface (*h* = 55 mm). Every surface consists of 44 composite magnetic profiles, each measured at a different tread’s width. Every circumferential profile consists of 1024 samples. First, the profile of surface is measured at w = 0, and the following with a w increase equal to 4.8 mm. Three surfaces are acquired this way. Composite surfaces 2 and 3 were subtracted from the composite surfaces of the first one. Finally, 2 × 3 composite surface of differences are obtained. The assumption is: If the highest standard deviation of every of 2 × 3 surfaces of differences is lower than admissible standard deviation of the sensor, then there is no detectable influence of the gear and equipment in this mode of operation.

## 4. The Results

### 4.1. The Noise in Different Phases of Operation

The sensors’ accuracy were assessed according to the value of standard deviation, which is measured on set of 1024 samples of *B_x_*, *B_y_*, and *B_z_*. During measurement, there was no wheel fixed on the shaft. Two experiments are performed. In the first experiment, the two electric motors are not powered in any way. In the second experiment, the motors were actuated as during the normal measurement. The sensor was placed at 5 locations determined in Table 3.

The results presented in Table 3 are convincing, showing that the operation of electric motors does not influence the magnetic flux density standard deviation. The differences are no greater than 0.3 × 10^−7^ T.

The next step is to compare the mean values of measured *B_x_*, *B_y_*, and *B_z_* components of B according to Formulas (5) and (6).

(5) $$\bar{B_{k}}=\frac{\int_{\varphi =0}^{\varphi =2\pi }B_{k}(\varphi )d\varphi }{2\pi },k\in \{x,y,z\}$$

(6) $$\bar{B_{k}}=\frac{\sum_{i=0}^{i=N}B_{ki}}{N},k\in \{x,y,z\}$$

where: *i*—index of particular *B_k_* value in profile, *N*—number of samples in circumferential profile (also the greatest index), *k*—designation of composite either *x*, *y*, or *z*; according to k the mean of profile consisting *B_x_*, *B_y_*, or *B_z_* values is calculated, φ—angle.

**Figure 6:**
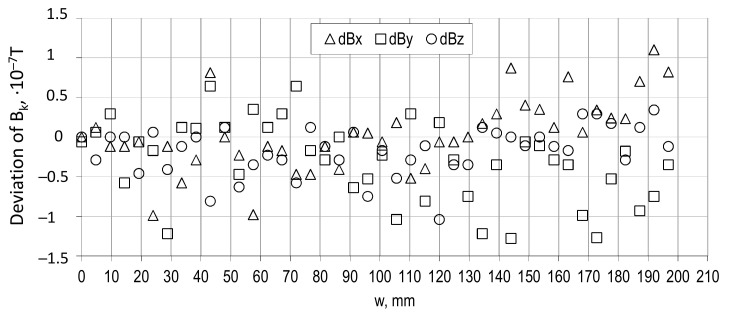
The discrepancy denoted as dB_x_, dB_y_, dB_z_ between mean values of *B_x_*, *B_y_*, *B_z_* of signals at perfect stop and during device operation as during measurement without wheel fixed for *h*_0_ = 450 mm and variable w (width of tire’s tread). The k index in *B_k_* means x, y, or z index as needed.

The discrepancy in the whole range of w (width of the tire’s tread) and *h_0_* is no greater than ± 1.5 × 10^−7^ T. Figure 6 is a vivid example of low discrepancy (lower than the sensor’s accuracy) of mean values of all **B** components at *h_0_* = 450 mm. The width (w) change between the following circumferential profiles is equal to 4.8 mm, since in the previous experiment, the increase of w was 100 mm.

Please note that the values of standard deviation and discrepancies are lower than noise level (2 × 10^−7^ T) predicted by the producer, which means that the above described rules and advice for measurement are efficient.

### 4.2. Repeatibility Test

**Figure 7:**
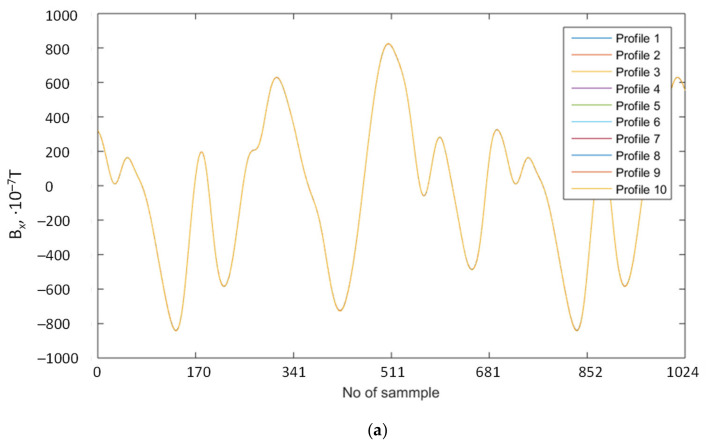

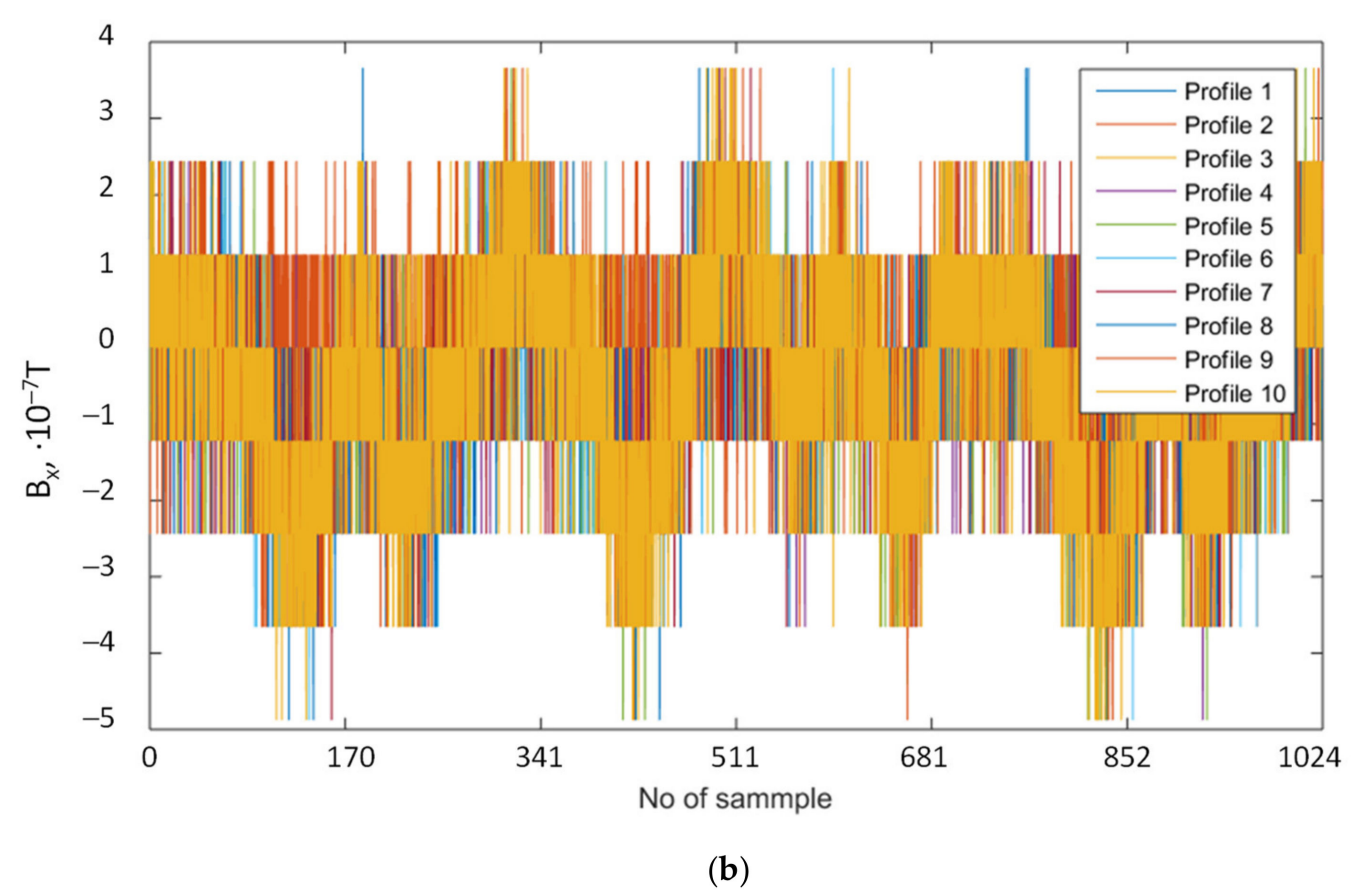
Magnetic profiles measured one after another in the same width of tread equal 100 mm for 205/60/R15/91 V tire (**a**) and differences between first and following profiles (**b**).

In this test, ten magnetic profiles are measure one after another in the same width of tread equal to 100 mm for the same tire fixed on 15′’ rim and for h equal 55 mm. The *B_x_* profiles are then plotted on the same graph in measurement order. In all cases of *B_x_*, *B_y_*, *B_z_*, and |B| the outcome is almost the same as depicted in Figure 7a—the last profile covered the profiles which are plotted earlier.

Nine magnetic profiles are calculated as a differences of Bx values of the same index in profile between first profile and the following profiles. Of course, the calculated differences between the same profile—the 1st and 1st is always zero. As it can be observed in Figure 7b, the maximum difference between profiles is ± 5 × 10^−7^ T, but the standard deviation calculated over the profile of differences never exceeded σ = ± 1.46 × 10^−7^ T. Almost the same values are obtained in every investigated case of profiles *B_x_*, *B_y_*, *B_z_*, and |B|. The maximal value of σ are never greater in all cases than σ_maxBx_ ± 1.46 × 10^−7^ T, σ_maxBy_ ± 1.61 × 10^−7^ T, σ_maxBz_ ± 1.85 × 10^−7^ T, σ_max|B|_ ± 0.9 × 10^−7^ T. This is again lower than sensor inaccuracy, and it proves that either measurement gear nor wheel manipulation during measurement (acceleration deceleration between measurements and speed control) does not affect the measurement outcomes.

### 4.3. “Sensibility” Test

The magnetic sensor (see det.16 on Figure 4) is mounted on a slide guide fastened to a pole (17, 18 on Figure 4) and is actuated (moved up and down by electric motor Figure 4, 15). The height of the sensor on the pole responds to width of tread (w) (see Figure 3) at which circumferential profile is measured. Many circumferential profiles form a magnetic plane (see Figure 3). In this experiment, the influence of gear responsible for (w) change, and magnetic plane measurement is tested. The test consists of measuring of 3 surfaces, one after another, of the same wheel. The *h* value is equal to 55 mm, and every circumference magnetic constituting magnetic surface is separated from another profile by a value equal to 4.8 mm. All measured surfaces are then subtracted from the first one, forming surfaces of differences. If any bias or increase of standard deviation occurs in surfaces of differences, then there is influence of the equipment (gear).

**Figure 8:**
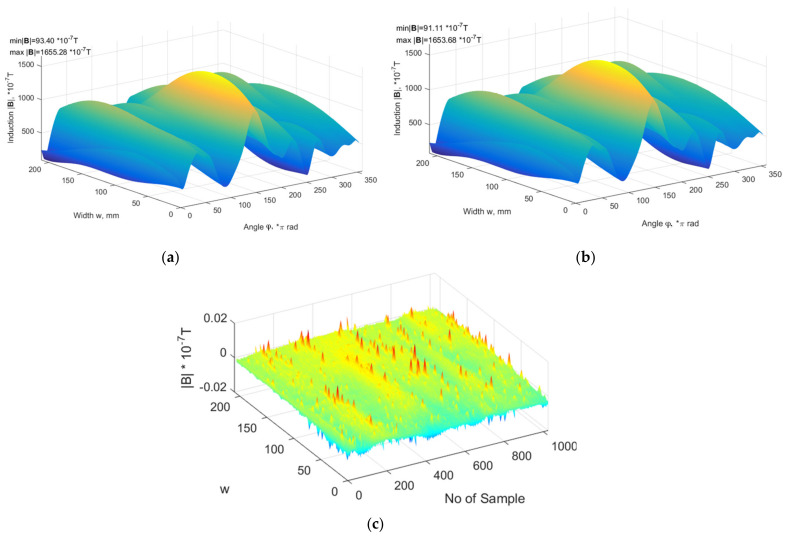
Magnetic profiles measured one after another in the same width of tread equal to 100 mm for R16/205/55 T tire (**a**)and (**b**), and differences between first and following profiles (**c**).

In Figure 8a,b, the measured 1st surface and 2nd surface are depicted. The shapes are almost identical. The extremal values min and max differ by ±2 × 10^−7^ T (see descriptions in left upper corners of Figure 8a,b), in this case indicating that there are some differences, but the surface of differences depicted in Figure 8c shows that the values oscillating in the range ±0.02 × 10^−7^ T (the smallest values in experiment), moreover, their distribution is random. For the other surfaces in the experiment, the values are also similar. The highest values of differences between the compared surfaces are as follows for *B_x_* ± 4.41 × 10^−7^ T, for *B_y_* ± 5.47 × 10^−7^ T, for *B_z_* ± 5.69 × 10^−7^ T, and for |B| ± 5.33 × 10^−7^ T. The standard deviation is at least 3 times smaller than the highest differences (the highest σ_max_ ± 1.93 × 10^−7^ T), but still smaller than that declared by the producer for the sensor uncertainty. This proves directly that there is no measurable influence of motors, gear, operation, or measurement procedure on results obtained by magnetic sensors, and indirectly, that the assumed requirements regarding gear design and parameters are correct.

## 5. Conclusions

According to many years of investigations, design activities, and experiment results, the following conclusions can be drawn out.

The materials used for sensor gear construction should not influence the magnetic field of the wheel. Ferromagnetic alloys must be avoided because of their high relative permeability, but austenitic steels, aluminum alloys, and brass, as well as artificial materials, can be used because of sufficient yield stress and hardness.

The acceleration of the shaft should be restricted to avoid relative movement between the shaft and wheel. The constant acceleration equal to 0.1 rad/s^2^ in this case is found to be sufficient.

The use of an electric motor with 1:62 reduction gear and timing belt gear of a ratio equal to 1:10 reduces the electrical current needed for rotational speed sustaining during measurement. The magnetic flux density produced by reduced currents in cables in this case is below the magnetic sensors’ resolution and accuracy.

Regardless of measuring technique (in time or in angle of rotation domain), the designer must decide whether to use the angular speed control of the wheel during measurement or not. The controller should be tuned as overdamped to fulfill the equation given in (2)–(4).

The accuracy and repeatability is the effect of all design decisions made in the areas of mechanical design, programing, electric and electronic systems, and also the operation algorithms. In this measuring device, constant magnetic flux density compensation algorithm was implemented, which lead to the achievement of a measurement of uncertainty at the level ±1.9 × 10^−7^ T.

The 3-axis anisotropic magnetoresistive HMC5883L can be used to measure the magnetic profile (1D) and magnetic surface (2D) in conjunction with gear designed according to rules pointed out in this article. This is because it is proven that there is no measurable influence of motors, gear, operation, or measurement procedure on the results obtained by magnetic sensor. The assumed requirements regarding gear design and its parameters are correct. The highest uncertainty obtained in accuracy and repeatability tests were:, Respectively, σ_max_ ± 1.93 × 10^−7^ T and at least ±1.23 × 10^−7^ T. Both results are lower than sensor accuracy and resolution declared by the producer.

Direct at the tire’s tread surface the sensor range should be few mT. The measurements farther from the range should be lower. As the measurement is usually done 33, 55, and 77 mm above the tread, therefore it is convenient when the sensor has a selectable range as the HMC5883L has. The resolution of the sensor should be at least 12 bit, the accuracy and sensitivity no lower than ±4 × 10^−7^ T. In this investigation, the stable level indications of amplitude appeared in amplitude spectra was 4–8 × 10^−7^ T. Typically, the range ±10^−5^ T and resolution are enough if the sensor is placed no farther than 250 mm from the tire’s tread.

At this precise moment, quite precise and repeatable measurement is a significant achievement in relation to other approaches. It makes the results reliable and the process’s influence on magnetic field change detectable.

The magnetic parameters of tire and wheel can be used for monitoring purposes, adding to work inspired by [29]. The magnetic parameters can be adopted for diagnostic of a steel belt in wheels performed on moving cars as well performed on a test stand. This will be a future direction of investigations undertaken by the author.

## Figures and Tables

**Table 1:** Relative permeability of selected materials used in wheels, tires, and instruments.

Material	$\frac{\mu }{\mu_{0}}\;[-]$		σ, MPa	Hardness
Ferrite steel	10	2300	300–900 Mpa	250 HB–35 HRC
Alumina AlMgSi 0.5 F25	1.000020	1.000030	200 Mpa	75 HB
Alumina AlCu_4_MgSi(A)	1.000020	1.000030	200 Mpa	75 HB
Stainless steel 316L	1.003	1.02	627 Mpa	79 HB
Brass	1.060		400 Mpa	70 HB
Rubber	1		22 Mpa	40 (Shore)
Teflon	1		23 Mpa	52–65 (Shore D)
Polyamide	1		83–103 Mpa	75–85 (Shore D)
Air	1.00000037		-	-

**Table 2:** Measuring device mechanical characteristics.

Parameter	Range	Comment
Wheel’s rims	12″ to 30″	Steel, light alloys rims can be used
Rotational speed of spindle	0.104 to 0.314 rad/s	(0.5 ÷ 1.5 rpm) Selectable, increment by 0.25 rpm
Max angular resolution	Up to 4096 samples per rot. ± 0.0015 rad	0.087 deg (selectable)
Circumferential resolution	0.25 to 0.6 mm	Depends on wheel’s free radius
Type of magnetic sensor	3D (3 Cartesian proj. of B)	Anisotropic, magnetoresist. HMC 5883L
Sensor dimensions	3.0 mm × 3.0 mm × 0.9 mm	Length × Width × Height
Range and resolution of magnetic sensor	From ± 1.5 × 10^−4^ T to ± 8.1 × 10^−4^ T, 12 bit	
Greatest std. dev. of 4095 samples measured at stop	±1.9 × 10^−7^ T	Max of std. dev. Meas. on *x*, *y*, and *z* axis
Distance to thread surface, *h*	10 to 300 mm	Extendable by additional fixture
Width of tread (*w*) at measurement	0 to 220 mm	Extendable by additional fixture, measured from top of fixing screw

**Table 3:** Measuring device mechanical characteristics. Standard deviations std(∙) of component profiles.

Sensor Location		Motors Off			Actuated Motors		
[mm]	[mm]	[× 10^−7^ T]			[× 10^−7^ T]		
w	h_0_	std(*B_x_*)	std(*B_y_*)	std(*B_z_*)	std(*B_x_*)	std(*B_y_*)	std(*B_z_*)
0	300	0.78	1.04	1.33	0.82	0.91	0.98
0	600	0.68	0.98	1.09	0.77	1.01	1.01
100	450	0.69	0.81	0.98	0.72	0.68	0.88
200	300	0.64	0.71	0.78	0.94	0.88	0.98
200	600	0.62	0.67	0.75	0.82	0.76	0.84

The *h*_0_ is measured from axis of rotation of wheel to the sensor’s location. The *h*_0_ definition differs from *h*. The *h* is the distance from the wheel’s tread to the sensor. The ω in all measurements was equal to ω = 0.107 rad/s (*n* = 1.03 rpm).

## Data Availability

Not applicable.
